# Hospice: Rehabilitation in Reverse

**DOI:** 10.4103/0973-1075.73640

**Published:** 2010

**Authors:** Senthilkumar Jeyaraman, Ganesan Kathiresan, Kavitha Gopalsamy

**Affiliations:** School of Therapeutic Sciences, Masterskill University College of Health sciences, Malaysia; 1 Clinical Physiotherapist, Malaysia

**Keywords:** End of life care, Hospice, Physical therapy, Rehabilitation in reverse, Review

## Abstract

Hospice care is about quality of life at a time when a person has an illness for which curative measures are no longer possible, and for which a physician has determined the patient has a life expectancy of about six months or less, a hospice program can support the process of death and dying in a compassionate way. A growing trend is to utilize physical therapy more frequently in hospice. Physical therapy has several vital roles in hospice care as follows: maximizing functional ability and comfort to enhance quality of life; assuring patient and care giver safety; helping people redesign their lives and life goals; providing support around physical, emotional and spiritual issues at the end of life. The purpose of this review is to provide 1) a description of hospice care, 2) an explanation of the roles of physical therapists in hospice care.

## INTRODUCTION

“Hospice” according to Webster is a refuge for a weary traveller. The current use of the term hospice to describe a concept of care for the dying patient conforms to the Webster definition. Throughout the world today, hospice is defined as the provision of a comforting and caring environment for the patient who has travelled through the maze of modern medical technology and has recognized that the limits of technology preclude a cure and perhaps even a comfortable existence. The patient may then choose to follow a palliative course of treatment rather than an aggressive course and enter what one hospice has named “the hospice way of living.”[1,2]

One of the most important cornerstones of a hospice program is a dedicated team of care providers. Physical therapists play a valuable role in the hospice team.[3] Physical therapy, or the provision of physical comfort through therapeutic measures of a physical nature (eg, heat, cold, and massage), is an old concept as hospice. Both the practice of physical therapy and the present day hospice in which it is used are evolved into multidimensional areas of health care. To explore these complex issues, this review focuses on the role of hospice in terminally ill patients and the role of the physical therapist in the hospice care.

## BRIEF HISTORY

The crusades provide some of the first accounts of hospice. Commonly operated by religious orders, these way stations provided shelter, food and spiritual care for pilgrims in a supportive, dignified setting. As these institutions developed, they became rudimentary medical centers.

In the middle of the nineteenth century, a hospice was formed in France by widows of cancer patients. Concurrently a similar institution was founded in Dublin, Ireland, by the Irish sisters of Charity. About 50 years ago, this religious group started St. Joseph’s Hospice in London. Soon, others were established in England under the guidance of Anglican and Methodist churches. Although cancer patients appeared to form the principal population, others diagnoses were also recorded, such as tuberculosis of the bone.

Currently, hospice is designated as a program that helps dying patients and their families to live through this final stage of life while receiving sophisticated holistic medical care. The hospice that has received the most public attention is St. Christopher’s Hospice in London, England. St. Christopher’s has served as the prototype for hospice care in England and the United States with a free standing unit providing inpatient care and a large home-care program. Dame Cicely Saunders, the medical director, has served as a spokesperson for hospice care and has provided much of the literature on the subject.[2]

## HOSPICE DEFINED

According to Markel and Sinon, a hospice is an autonomous, centrally administered program of coordinated in and outpatient services. This physician directed program of health care delivery employs a multifaceted approach: narcotic and non-narcotic analgesics are used in physical symptom control, and the interdisciplinary hospice team provides psychologic, sociologic and spiritual services as needed. The patient and family is the primary unit of care and services are available on a 24-h, seven-day-a-week basis. Hospice services are also available during the period of bereavement. Patients are usually accepted on the basis of health needs, rather than the ability to pay.[4]

### Symptom control

Hospice care is directed primarily at symptom control, not at disease control. Perhaps the most publicized aspect of hospice care is control of pain. The pain control is only one of the measures taken to maintain the patient’s quality of life. Hospice care also takes into consideration concurrent stressful symptoms, such as loss of appetite, nausea, vomiting, hiccups, itching, labored breathing, coughing, insomnia, depression, anxiety, incontinence, constipation and difficulty in swallowing. Eliminating the pain and physical symptoms of the patient are only two aspects of the hospice concern for maintaining the quality of life.[2,5]

### Interdisciplinary team

Providing an interdisciplinary-care team for the family-patient unit is another feature of the hospice approach. This team includes physicians, staff nurses, home health care nurses, social workers, psychologists, physical therapists, occupational therapists and trained volunteers. Although patients and family members are encouraged to relate to any and all team members regardless of formal role designation, the involvement of team members varies from case to case depending on the needs of the patient.[6]

### Family-patient care units

The unit of care is the patient and his family. Zimmerman emphasized that the family also experiences stress with the patient’s terminal illness.[7] Thus, the hospice seeks to develop the insight of the family and patient while giving support. Krant quoted an earlier study by Hampe in which spouses related some of their needs in dealing with a dying loved one.

“To be with the dying person at the moment of death; to be helpful to the dying person; to be assured of the person’s comfort; to be daily informed of the patient’s condition; to be privately informed of the impending death; to be able to vent their own emotions; to be comforted and supported by the family; and to feel acceptable and support from the medical staff.”[8]

Our health-care system appears to have some difficulty in providing this type of supportive care; 75% of those interviewed in the Hampe study did not find these needs met.[8]

### Staff coverage

Hospice care is both an inpatient and an outpatient program. A commitment to 24-h, seven-days-a-week attention exists whether the patient is being cared for at home or in the hospice setting. Hospice services are also available during the period after the patient’s death. The medical-care team maintains an ongoing relationship with the family after the death of the patient. Hospice care continues after the death of the patient with emotional support and practical follow-up provided for the family members.[2]

### Philosophy

Death in perspective as part of the life cycle. Hospice rules and regulations for inhouse service format have an unheared of flexibility. Visitors may arrive at any hour or stay to prepare a favourite meal for the patient. Children and pets are welcome as are personal possessions belonging to the patient. Patients can spend a day or evening or both outside the hospice with a friend or family member or even go home for a weekend.[2]

## HOSPICE GOALS

The three goals of hospice as stated by Rizzo and Rizzo are

1. Relieving the patient-family unit of pain (patient with chronic physical pain and family with the social pain of helplessness)
2. Easing emotional, social, economic and spiritual stresses
3. Assisting the family in overcoming the stress and emotional pain felt after the patient’s death.[9]

## HOSPICE VERSUS HOSPITAL INPATIENT CARE

In previous studies, the quality of care for dying patients in general hospitals was analyzed, in which some problems were identified. They were staff avoidance of dying patients[10–14] inadequate symptom control,[15] lack of provision of basic nursing care,[12] poor communication,[16] a focus on physical needs at the expense of psychological needs[10–12] and being too busy to provide adequate care.[16] Some comparisons of hospice and hospital inpatient care have suggested that higher standards are obtained in hospices.[17–21]

In two studies of St Christopher’s Hospice, London,[18–20] Parkes found that spouses of hospice patients were less likely to report having experienced anxiety during period of care. They also spent more time at the hospice and were more likely to talk with other patients and visitors than were spouses of hospital patients.

In the USA, Kane *et al*.’s randomized controlled trial of hospice care found some decrease in carer’s anxiety and more satisfaction with involvement in care among hospice group carers.[22]

## HOSPICE CONCEPTS, APPROACHES AND CORRESPONDING PT CONCEPTS, APPROACHES

Koff gave outline about the basic values of hospice and its corresponding physical therapy approaches which was constructed appropriate to hospice care. The basic values are shown in Table 1, which allows physical therapy practitioners to orient themselves to a possible new paradigm for involvement with geriatric terminally ill clients and their loved ones.[23]

**Table 1:** Hospice concepts and approaches and corresponding PT concepts and approaches

Hospice values and approaches	Physical therapy approaches
The basic regard for the recipient of care	Client centered
Acceptance of death as a natural part of living	Human development
Consideration of the entire family as the unit of care	Family dynamics and systems thinking
Maintenance of the patient at the home for as long as possible	Environments of care; environments modifications and adaptations; caregiver training
Assistance for the patient attempting to assume control over his or her own life	Psychosocial and psychospiritual enhancement via provision of improvements or adaptations in function
Instructions for patient self-care	Health education: home, hospice, or in bed programming ideas to maximize function
Reduction or removal of pain and other distressing symptoms	Pain management, strengthening when possible, energy conservation, work simplification, complementary techniques
Comprehensive provision of services by an interdisciplinary team	Team approach
Total not fragmented care	Holistic thinking and clinical reasoning
Continuity of services after death	Grief, bereavement, and planning for more positive physical and mental health for survivors

## THE VALUE OF PHYSICAL THERAPY IN HOSPICE

“There is always something that physical therapy can do to provide function or comfort to the patient who is terminally ill,” says Steve Gudas, PT, PhD, who has dedicated his 31-year career to oncology physical therapy.[3] Physical therapy for patients receiving hospice care is directed at achieving symptom control, maximizing remaining functional abilities, providing caregiver education, and contributing to interdisciplinary team communication. In hospice care, physical therapists assist patients in maintaining their self-identity, waiting “actively” for death, achieving a comfort level and confidently using their remaining abilities as the gradual reduction in functional abilities, roles, and expectations occurs.[24] The interventions provided by therapists are directed to six facets:

### Delivering direct patient care

Physical therapists play a key role on the hospice care team. Physical therapists provide such services as:[3]

1. Pain management and relief
2. Positioning to prevent pressure sores, decrease pain, help to prevent contractures, and aid in breathing and digestion
3. Endurance training and energy conservation techniques
4. Gait training, stair climbing, transfers and safety instruction
5. Therapeutic exercise
6. Edema management
7. Equipment recommendation, modification and training
8. Home modification

### Educating the patient-family care unit and fellow health professionals

Although the physical therapist often provides “hands-on” treatment for the hospice patient, more prevalent role is that of educator. Education can take three directions that may occur concurrently.[3]

1. Obviously, teaching the patient to move efficiently and safely is a patient-therapist interaction
2. Another phenomenon occurs, as the therapist instructs the patient, the patient’s care giver, the family, also receives instruction. This is particularly true with home-care hospice programs if the other members of the interdisciplinary team are present, they also receive instruction
3. Finally, the physical therapist assumes the role of counsellor as does each member of the team. This counselling may be on a formal or informal basis with the patient, the family, or another health professional. Skills in communication are particularly important in this role. The art of active listening demands as much from a therapist as being able to express oneself with clarity and sensitivity

### Functioning as a team member

Physical therapists and other professionals in hospice must demonstrate not only well-developed clinical skills but also the ability to communicate effectively and facilitate team interaction and innovate extemporaneously. Interdisciplinary team membership exacts all of these skills from each professional. They could be identified as the foundation on which hospice depends. For this foundation to support a strong program, the formation and function of an interdisciplinary team must be thoroughly understood by each member.[3]

### Restoration of dignity (a shift in our thinking)

In consideration of all the avoidance of injury, safety hazard resolution, pain relief and reassurance therapy provided, Miriam Frost, PT, BS believe in restoring the sense of self of a patient, which had an incredible influence on their quality of life, decreased the frequency of nursing and social worker visits and avoided the potential for injury in the home for the patient and physical strain on their care givers.[25] For hospice work, it is imperative that we utilize the strength of our presence and the solid base of our professionalism to enhance the dignity and quality of life that promotes a healthy life until death.[26]

### Psychosocial spiritual support

Spirituality is an important component of hospice work and the physical therapist who works in hospice care must be ever mindful of the need for spirituality in both assessment and intervention. Spirituality incorporates meaningful occupational engagement. This is one of the leading differences in both environments of care (eg, hospital system versus hospice framework) and in practice for physical therapy practitioners.[26]

Research suggests that older patients receiving hospice care may benefit from intervention grounded in issues related to “family,” spirituality, outlook on mortality, and meaningful physical activity.[24]

### Therapist self care

The personal awareness and need for support of the therapist working with the dying patient warrant attention to promote the best care as well as the health of the therapist. It is difficult to help the dying if the therapist has not acknowledged his or her own uncomfortable fears about death.[27] Our faces and body language reveal those emotions, so it is essential to develop a practice of reflection and self-examination to explore those issues. Honesty and introspection into our own dying and therefore better serve the patients in hospice who are in our care.[28]

## REHABILITATION IN REVERSE

The more usual pattern in hospice and an unfamiliar concept for the therapist to apply is that of “rehabilitation in reverse” as identified by Briggs,[29] which includes exploring the process of functional adaptation and occupational engagement on a daily basis. Throughout each phase of decline during the dying process, new or adapted skills and abilities must be learned by the patient, as well as the care givers, to maximize functional independence and safety. The therapist has the knowledge and skill to assess current status; teach appropriate techniques such as transfers, bed mobility and positioning to decrease pain; collaborate with patients on relaxation techniques; adapt activities of daily living and mobility; and instruct in the use of needed equipment. Other team members may provide some basic instruction but the therapist should be consulted for further care. Therapists provide skilled therapy service that achieves the hospice goal of promoting safety, independence, meaning and quality of life, despite the physical and mental decline which is expected.

For example, the physical therapist might have a patient who has a brain tumor and is unsteady. The patient has been walking without a cane, and now the therapist has to teach him how to use a cane and teach family members to assist with balance. Then, a week or two later, the therapist need to fit him with a walker and teach him to use it. A month later, therapist needs to teach transfers from bed to a wheelchair. And one week later, the therapist might be positioning for pressure relief. As the patient’s health is declining, there is always some level of skilled care that the therapists can provide.[3]

## LIABILITY

The services that PT’s provide to patients and caregivers through hospice programs are within the scope of physical therapy practice and should therefore be routinely covered within the scope of physical therapy practice and should therefore be routinely covered within a physical therapist’s liability insurance policy. However, physical therapists are well advised to practice good risk management and before entering a new practice arena, to research and understand what risks are involved and take steps to ensure that they are adequately protected.[3]

## REWARDS

While there is great opportunity for more physical therapists to make a difference in the hospice setting, this is clearly an area of practice that is not for everybody. Becoming involved in a family’s grief process can certainly be emotionally draining. But physical therapists with experience in this area of practice overwhelmingly report that the rewards of hospice care are indeed rich.[3]

Says Steve Gudas, “The spirit, the strength, the courage, the resiliency, and the determination that people have toward the end is amazing to witness. And it is a privilege to play a role in the process. It is actually life affirming.”[3]

Adds Nicole Gergich, “It is gratifying to connect with a person at this time in his or her life. We can offer the patient control, independence, and dignity. Yes, it is sad that the person is dying. But if I am the one who can help, Iam glad to have been part of it. It is rewarding and reinforces to me how very important my job is.”[3]

## HOSPICE AND PHYSICAL THERAPY RESEARCH

De Rave in his study cites terminally ill patient’s vulnerability, dependency and compromised autonomy and makes the case that no research can be justified with dying people.[30] Palliative care research is not simply curiosity-based experimentation, they make a strong case for the critical role of research in developing proven therapies that can provide relief from pain and the many other symptoms with which palliative care patients suffer greatly.[31] On the other hand, many palliative care researchers think that the opportunity to participate in research protocols can be a positive and meaningful experience for patients.[32–34] Currently there is a beginning body of research in the area of hospice, terminal care, and end of life care in physical therapy.

## SUMMARY

As hospice organizations seek to raise awareness about their programs, there is also opportunity for physical therapists to raise awareness about the valuable and unique role they bring to the hospice team. “PTs have not traditionally been a routine service provider in this setting,” says Nicole Gergich, PT, President of APTA’s oncology Section.[3] The role of the physical therapist in hospice care is different from the role the therapist plays when a member of a rehabilitation team. It is like transitions from rehabilitation to end of life care. In working with hospice patients, therapists must, for the most part, change from a controlling role to one of listener and problem solver.

## CONCLUSION

In an ever-changing health care environment, new practice arenas continue to emerge for physical therapists. Hospice care is one among them. Though this practice area is underserved, it is alive with possibilities and opportunities. This review constitutes a first step in evaluating the role of physical therapy in hospice care. Further randomized controlled studies are necessary to validate the efficacy of physical therapy in hospice care.

“You matter because you are you. You matter to the last moment of your life, and we will do all we can not only to help you die peacefully, but also to live until you die,” Dame Cicely Saunders.

## References

[1] (1982). Annual report of hospice of South-eastern Michigan.

[2] Toot J (1984). Physical therapy and hospice – concept and practice. Phys Ther.

[3] “Emerging PT Practice: Hospice care” (“Professional Resources,” “Practice,” then “Emerging PT Practice information”). http://wwwaptaorg.

[4] Markel W, Simon V (1978). The hospice concept.

[5] Hospice CK (1979). A Prescription for terminal care.

[6] Worby CM, Blackman KS, Schneider J (1978). Hospice care: Current status and future prospectus. Patient Couns Health Educ.

[7] Zimmerman JM (1979). Experience with a hospice care program for the terminally ill. Ann Surg.

[8] Hampe SO, Krant MS (1978). The hospice movement. New Engl J Med.

[9] Rizzo RF, Rizzo L (1978). Hospice program. NY State J Med.

[10] Buckingham RW, Lack SA, Mount BM, MacLean LD, Collins JT (1976). Living with the dying: use of the technique of participant observation. Can Med Assoc J.

[11] Conboy-Hill S (1986). Psychological aspects of terminal care: A preliminary study of nurses’ attitudes and behaviour in a general hospital. Int Nurs rev.

[12] Mills M, Davies HT, MacRae WA (1994). Care of dying patients in a hospital. Br Med J.

[13] Glaser BG, Strauss AL (1965). Awareness of dying.

[14] Sudnow D (1967). Passing on: The social organisation of dying.

[15] Hockley JM, Dunlop R, Davies RJ (1988). Survey of distressing symptoms in dying patients and their families in hospital and the response to a symptom control team. Br Med J.

[16] Addington Hall J, Mac Donald LD, Anderson HR, Freeling P (1991). Dying from cancer: The views of bereaved family and friends about the experiences of terminally ill patients. Palliat Med.

[17] Hinton JM (1979). Comparison of places and policies for terminal care. Lancet.

[18] Parkes CM (1979). Terminal care: Evaluation of in-patient service at St. Christopher’s Hospice. Part I: views of surviving spouses on effects of the service on the patient. Postgrad Med J.

[19] Parkes CM (1979). Terminal care: Evaluation of in-patient service at St. Christopher’s Hospice. Part II: Self assessments of effects of the service on surviving spouses. Postgrad Med J.

[20] Parkes CM, Parkes J (1984). Hospice versus hospital care re-evaluation after ten years as seen by surviving spouses. Postgrad Med J.

[21] Seale CF (1991). A comparison of hospice and conventional care. Soc Sci Med.

[22] Kane RL, Klein SJ, Berstein L, Rothenberg R, Wales J (1985). Hospice role in alleviating the emotional stress of terminal patients and their families. Med Care.

[23] Koff (1980). Hospice: A Caring community.

[24] Mackey KM, Sparling JW (2000). Experiences of older women with cancer receiving hospice care: significance for physical therapy. Phy Ther.

[25] Frost M (2001). The role of physical, occupational and speech therapy in hospice: Patient empowerment. Am J Hosp Palliat Care.

[26] (2004). Pizzi MA and Briggs R (2004): Occupational and Physical therapy in Hospice. The facilitation of meaning, Quality of life and well-being. Top Geriatr Rehabil.

[27] Rimpoche S (1992). The Tibetan Book of Living and Dying.

[28] Sharp J (1996). Living Our Dying.

[29] Briggs R (1999). Models for physical therapy practice in palliative medicine. Am Phys Ther Assoc Home Health Sect Q.

[30] De Rave L (1994). Ethical issues in palliative care research. Palliat Med.

[31] Mount BM, Cohen R, MacDonald N, Bruera E, Dudgeon DJ (1999). Ethical issues in palliative revisited. Palliat Med.

[32] Janssens R, Gordijn B (2000). Clinical trials in Palliative care: An ethical evaluation. Patient Educ Couns.

[33] Addington –Hall J (2002). Research sensitivities to palliative care patients. Eur J Cancer Care (Engl).

[34] Hudson P (2003). The experience of research participation for family care givers of palliative cancer patients. Int J Palliat Nurs.

